# Nocebo Effects and Negative Suggestions in Daily Clinical Practice – Forms, Impact and Approaches to Avoid Them

**DOI:** 10.3389/fphar.2019.00077

**Published:** 2019-02-13

**Authors:** Ernil Hansen, Nina Zech

**Affiliations:** Department of Anesthesiology, University Hospital Regensburg, Regensburg, Germany

**Keywords:** physician-patient communication, informed consent, nocebo effects, therapeutic communication, nonverbal suggestions, natural trance, negations

## Abstract

Medical situations are hot spots in the life of a patient with potentially long lasting effects arising from the use of either negative expressions or encouraging statements, or the lack of empathy or a positive physician-patient relationship. Health care personnel should be aware of and evaluate what patients are exposed to, hear and see. Knowing more about the effects of nocebos and negative suggestions, combined with increased attention to these matters, provides the basis for better recognition of detrimental influences in their own clinical environment and to be able to avoid, stop or neutralize them. After anamnesis patients should not be left with a focus on a negative past, but shifted to positive experiences prior to their illness, or to positive expectations in the future following surgery and rehabilitation. For example, after examining an injured leg the doctor should not turn to the computer for documentation unless he has shifted the patient’s focus on the other, unimpaired leg. “Is that painful too? No? Good! Can you feel that? Yes? Perfect! Can you bend that knee, move these toes? Great! That’s good.” This example draws attention to the fact that negative effects (discussed in the following) substantially are dependent on the focus of the patient and thus can be affected by focus shift and distraction. Patients, their symptoms and their healing are negatively affected by the omission of placebo effects, by nocebo effects and by negative suggestions.

## Omission of Placebo Effects

Health personnel have an impact on patients and therapeutic effects even when they do not talk, or because they do not talk.

### Lack of Communication

**FIGURE 1:**
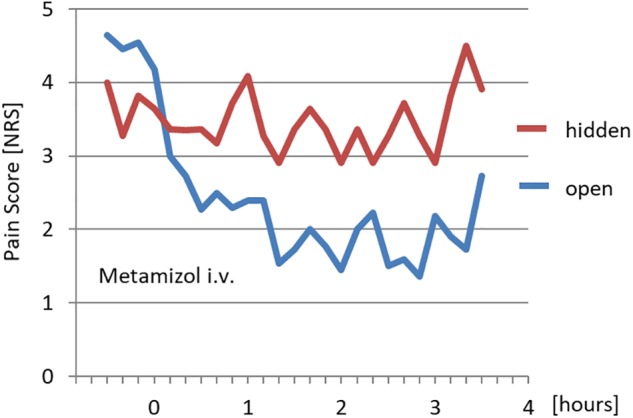
Effect of open and hidden application of metamizol on pain [adapted from Benedetti (2013)].

The analgesic effect of metamizol was significantly lower when the intravenous application was not announced (Benedetti, 2013) (Figure 1). The same holds for other analgesics including morphine (Price et al., 2008), where we think we know exactly how it works. In the “hidden” therapy only the drug itself exerts its purely pharmacological action, while in the “open” therapy the expectation of the patient adds the placebo effect, and thereby the therapeutic effect is significantly increased. While according to the classical paradigm a placebo effect is evaluated by applying an inert drug or a sham intervention, in the open/hidden paradigm therapy is not withheld from the patient and the placebo effect arises out of the difference between the effect of usual therapy (open = drug and expectation effect) and the treatment without announcement (hidden = solely drug effect) (Finniss and Benedetti, 2005). Strong placebo effects are also reported for other drugs (Hrobjartsson and Gotzsche, 2004) as well as for surgical procedures (Jonas et al., 2015). Especially with pain relieving interventions like spinal discectomy, knee or shoulder arthroscopies, and percutaneous procedures like stent implantation sham surgery reaches over 70% of the verum effect (Jonas et al., 2015). This means that the placebo effect significantly contributes to most therapeutic interventions (Evers et al., 2018). Therefore, announcement and communication of the treatment must be an important integral part of any treatment. In order to utilize the placebo effect, any application of a drug, even to a sedated or comatose intensive care patient (see later), should be accompanied by communication. The same holds for any other intervention like application of a bandage, positioning of a patient, or physical therapy. In the terminology of following recent expert consensus (Evers et al., 2018) all health changes that result after administration of an inactive treatment are named placebo and nocebo response, while “placebo and nocebo effect” refers to the changes attributable to placebo and nocebo mechanisms, including the neurobiological and psychological mechanisms of expectancies. Major implications for clinical practice are seen in the use especially of such effects without any “placebo,” i.e., an inert drug or a sham intervention, which also can be termed “placebo-like effects” (Benedetti, 2008). Realizing the important contribution of these placebo effects to therapy, we should start improving our communication to elicit and enhance the necessary respective positive expectations in the patients (Hansen et al., 2017). The other way around: Any doctor, nurse, paramedic or physical therapist who does not accompany his or her intervention with positive expressions that create positive expectations, will have a diminished impact by withholding placebo effects and potentiation of therapeutic efficacy. In most studies of placebo effects the exact wording is not reported, but wide variation in the effects hint at the impact of the specific terms used and we are only beginning to learn how to improve our therapeutic efficacy that way.

In this context, announcements with the inappropriate wording can decrease or abolish the therapeutic effect, or even result in the opposite of the desired effect. Dentists that announced nitrous oxide as a drug that enhances sensitivity abolished the analgesic effect (Dworkin et al., 1983). A muscle relaxant, announced as a stimulating agent, increased tension instead of causing relaxation (Flaten et al., 1999). Ipecac, a drug very successfully used to induce vomiting (e.g., after ingestion of toxins), reduced nausea and vomiting in pregnant women when announced as an antiemetic (Wolf, 1949). Accordingly, use of the wrong wording can interfere with our desired therapeutic goals.

### Lack of Meaning

The most important factor for increasing the effectiveness of the potentiation of therapy through our words is the addition of meaning to the transmitted information. “Let me give you a blanket. I put on a strap” said during preparation of a patient for surgery meets the need for announcement of any intervention but is restricted to information. By phrasing it as “Let me give you a blanket for your comfort. I put on a strap for your safety” meaning is added, and the induced positive expectation can exert placebo effects of feeling comfortable and safe. Moerman, who proposed to replace the term “placebo effect” with the term “meaning response,” stated that people are not responding to placebos but to meanings (Moerman and Jonas, 2002).

Moreover, meaning and positive expectations can be induced aside from specific occasions. In an “essential communication” the general basic needs of the patient can be addressed, whether for transportation to the hospital or the radiology department, during gastroscopy or during surgery under regional anesthesia. The topics that should be covered and the words a patient needs to hear can be derived from the human basic psychological needs (Grawe, 2000) and from traumatic stressors (Wilkinson et al., 2017), i.e., the factors that have been demonstrated to be the cause of development of posttraumatic stress disorder (Table 1). These topics, namely company, contact, comfort, control, care, information, instruction, respect, safety and healing, should be addressed repeatedly and in various, individual expressions and words. Those are the words a patient in need should and must hear. Company, for instance can be expressed by “I am here for you.” It can be increased by “we are here for you” and extended in time “and we’ll stay with you.” And further: “And where we take you other staff will take over and care for you.” The assurance of company and care can be amended with positive suggestions like “I will not move from your side until you have come through this well.” This wording evoked a thank-you card from a patient: “You surely have noticed my anxiety before that operation, but your holding my hand and your words “I will not move from your side” have really calmed and soothed me,” demonstrating the strange patient’s fear that the anesthetist would be leaving after induction of anesthesia. But this is what a doctor must face and react to: not hypothetical but real fears of patients albeit sometimes unexpected.

**Table 1:** Derivation of the topics for a positive “essential communication” with wide applicability.

Basic Psychological Needs	Traumatic	Topics of
(according to K. Grawe)		“essential communication”
Relationship and	Abandonment	Company
Belonging	Inability to express oneself	Contact
Pleasure Gain and	Pain, Suffering	Comfort
Prevention of Displeasure		
Orientation and	Chaos	Information
Control	Dependence	Control
	Helplessness	Instructions
Self-Esteem and	Degradation	Respect
Self-Protection	Fear, Threat	Safety
	Injury	Care, Healing

Accordingly, an introduction could be: “Hallo, I am Dr. X, and we are a whole team responsible for your comfort and your safety. We will stay with you and take care of you until you have successfully come through this.” Each therapist has to find his own words to address these topics and be authentic. All topics should be covered, because the actual and most essential need of the individual patient is sometimes difficult to assess. Each topic should not be addressed only once, but over and over again. This is a concept of exchanging nocebo effects by placebo effects, as feelings of abandonment or fear, for example, can be seen as negative expectations which, by assuring company and safety, are turned into positive ones. Applying this concept during the transport of emergency patients to the hospital, a better outcome was reported in the “Kansas Experiment” (Jacobs, 1991). The same principle of communication is the basis for taped suggestions during general anesthesia that can result in reduced pain and nausea, and saving of medication (Rosendahl et al., 2016).

## Nocebo Effects

Negative conditioning and negative expectations are common in medicine (Häuser et al., 2012).

### No Placebo Without Nocebo

**FIGURE 2:**
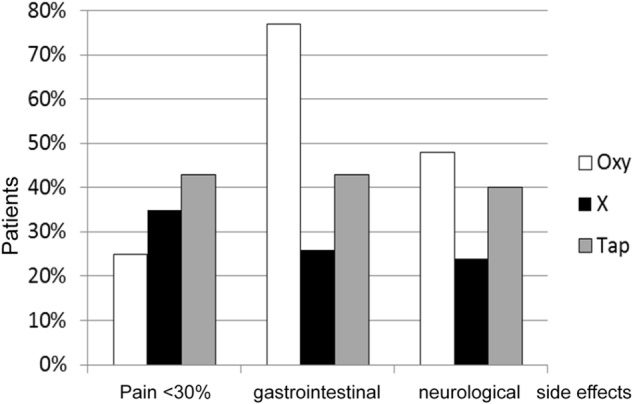
Effects and side effects of three “strong analgesics” [adapted from Afilalo et al. (2010)]. Oxy = Oxycodone X = Placebo Tab = Tapentadol.

When asked which of three strong analgesics (opioids) with known effects and side effects they would choose, most doctors would favor those shown in the middle column in Figure 2, because of their analgesic effectivity and moderate side effects. Actually, it represents a placebo. Doctors should realize that they cannot pick just the positive effect, i.e., the augmentation of their therapy by positive expectations, nocebo effects always go along with it. Looking at oxycodone in Figure 2 not all of the gastrointestinal and neurological side effects like constipation and dizziness stem from the drug, but to a great degree from the accompanying nocebo effect (Afilalo et al., 2010). This can be extrapolated to the realization that quite a number of patients treated for side effects did not get their problems as a result of surgery or drug administration but from nocebo effects that the treating physician has generated. Those symptoms are not imagined but real. In the end it is unimportant whether leukocytes were activated to release cytokines in an infected wound, leading to vasodilation, pain and edema, or by the expectation of an inflammation (psychoneuroimmunology). The results are identical and indistinguishable. In almost every placebo-controlled study there are side effects and drop-out of patients because of unbearable discomforts (Häuser et al., 2012; Howick et al., 2018).

### Clinical Examples

A wide spectrum of clinical examples and experiences add to the evidence for a rather negative medical environment patients commonly are exposed to. “You are a risk patient”: will predispose this patient for years. “I wouldn‘t be surprised, if this will stay a burden for you.”, “For elderly patients with such a nystagmus it usually is no big problem. But at your age?! You never will get accustomed to that!”, “You won‘t be able to …,” “You‘ll feel like run over by a truck.”, “You‘ll hurt like hell!”, “You are a walking time bomb.”, “Let me know when you start hurting.”, “You can have a heart attack or worse any minute.”, “You must take care that you don’t end up in a wheel-chair someday.”, “With these few contractions for some women it takes more than a week. You never know when will it come (childbirth).”, “When old people develop such a tinnitus it‘s not so bad, but at your age? You’ll never get used to it.”, “If you think this pain was unbearable just wait till you wake up after the operation – it will be 100 times worse!” or “It’s all over now!” are bad prophecies (Lown, 1996; Hansen and Bejenke, 2010; Faymonville et al., 2011; Zech et al., 2014). Drug side effects are much more common when the patient is informed (Häuser et al., 2012). Manifestation and extend of the induced side effect are dependent on the content of words with negative connotation (“severe headache,” of positive suggestions (“that we can treat), and of meaning (“This could put heavy strain on you for quite some time.”).”

In addition to comments from health care providers, expectations can also be influenced by fellow patients (observational learning) (Vögtle et al., 2016), media (Faasse et al., 2012), and visitors (“good willers”) (Savulescu et al., 2006). Words and situations in the waiting areas can predispose to bad experiences. A patient distorted with pain leaving the treatment room of a dentist sensitizes the patients in the waiting room. Reports in newspapers, magazines, radio and television affect acceptance and side effects of a vaccination (Faasse et al., 2017). Relatives and friends with their strong impact can hurt by thoughtless comments (e.g., mother: “How could you do this to me!”), and even well-meaning comments (e.g., “You don‘t hurt, do you”) can set negative expectations with expressions of caring concerns (e.g., “Hopefully you don‘t catch a hospital-acquired infection!”) and weaken the patient (e.g., “Be happy that you haven‘t lost your leg. You must accept your disability!”) (Savulescu et al., 2006).

### Neutralizing Negative Expectations

Negative experiences in the past are often extrapolated by the patient to the present (““I am …,” with me the case is …”) and into the future (“This will happen again”), leading to negative expectations with nocebo effects and a high chance that it really will happen again (Benedetti, 2013; Zech et al., 2015a; Hansen et al., 2017). Existing negative programming can be recognized when the patient describes his condition and symptoms with the word “always.” “I always have this terrible pain in my back when I get up in the morning.” This negative expectation can be disrupted by verbal mirroring and exchanging the word “always” with “most of the time” or by “often.” “Oh, I understand, you often have back pain when you get up in the morning.” This draws the focus away from the problem to exceptions and a solution, and changes the therapeutic approach from problem-oriented to solution-oriented. “What was different, when you woke up without pain?” “I had enough sleep.” “So you should do this more often.” Constructive w-questions instead of questions with yes-or-no-answers can produce a possible starting point for a change. Another option is to leave the symptoms in the past, where they actually belong. “I always have to choke when I am at the dentist.” “Yes, so in the past (or “up to now”) you always had to choke.” The fundamental common principle is to break up the idea of a fixed future and free the view to alternative possibilities. Similarly, restriction like “I cannot” should be overcome with a “not yet.” “I cannot stop smoking.” “I see, you have not yet found a strategy to stop smoking.” (Prior, 2011).

“Last time after surgery I felt so nauseous and had to vomit” means that the patient expects the same to happen this time again. The answer could be “Wouldn‘t it be great, if this time it is different. And there are good reasons …” or “Oh yes, I met quite some patients telling me that and then had surgery and anesthesia without.” And similarly in an emergency: “Will I die?!” or “Will I be able to move my legs again?!” reflects the expectation focus of a patient on the worst. “The trauma lies behind. Only the next hours and careful examinations and diagnostic procedures will allow us to see which of the various possibilities will remain and to answer your understandable question. But in any case, be sure that we all are here for you and give our best for your best outcome.” The solution for negative expectation is not to negate the problem, or lie, or whitewash, but to take the patient serious and open up the perception of other possibilities (Zech et al., 2014). Since any option has a certain probability, describing multiple possibilities decreases the probability of any given one, and thereby reduces also that of the expected negative one (Hansen et al., 2017).

### Avoiding Generation of New Negative Expectations

A rich origin of negative suggestions inducing negative expectations and nocebo effects results from risk information provided in order to receive informed consent (Zech et al., 2015a,b). Any symptom can be induced or worsened by inappropriate communication about it. After warning for gastrointestinal symptoms from aspirin the incidence of this side effect was six times higher than without the warning (Myers et al., 1987). Incidence of erectile dysfunction increased from 3 (“medication for the heart”) to 16 (“beta-blocker”) and 31% (“beta-blocker with possible side effect erectile dysfunction”) depending on the extent of information (Silvestri et al., 2003). While post-spinal headache is common after lumbar puncture and usually affects every other patient, it developed only in every tenth patient when information about this side effect was not communicated (Daniels and Sallie, 1981). Nevertheless, the conclusion by those authors that “patients should not be told to expect a headache as this may be a self-fulfilling prophecy” cannot mean omission of risk information, but requires a reconsideration of the way we present it. In addition, harsh risk information can destabilize circulation, increase anxiety and stress, both strong predictors for a bad outcome, and even keep patients from a necessary therapy (Zech et al., 2015a; Usichenko et al., 2016). A structured literature search in PubMed with the terms “(informed consent) AND nocebo” resulted in 27 suitable articles (Wells and Kaptchuk, 2012; Howick, 2012; Faasse and Petrie, 2013; Cohen, 2014; Heisig et al., 2015; Wilhelm et al., 2018).

However, elimination of risk information to achieve informed consent is not an option. A solution is the combination of the negative signals of risk information with positive aspects, such as the benefits of the therapy, the prophylaxis that is undertaken to prevent potential side effects, the monitoring for fast detection of a developing side effect, the resulting improved chances for successful treatment and reversal of the symptom, and suggestions for the patient’s own contribution to a positive outcome (Seemann et al., 2015a,b). “We will carefully disinfect the skin where we do the surgery to prevent wound infection” could be the risk information about wound infection. “The ECG-monitoring would immediately tell us should your diseased heart develop some arrhythmia, so we can start immediately with appropriate treatment.” is risk information about arrhythmia with the addition of the option of fast intervention. “If you repeat the breathing exercises I showed you often enough, you can contribute to the prevention of pneumonia” gives patients motivation and self-control. In a recent study we found objective proof of this concept (Zech et al., 2019). We measured effects of communication in the medical context on maximal arm muscle strength by dynamometry and observed significant weakening after risk information with regard to a pain catheter including infection, allergy, vessel and nerve injuries. The reduction in muscle strength was absent when the risk information was given together with the benefits of the treatment, like less need for taking pills, more comfort, and the possibility of a shorter hospital stay. Moreover, this fulfills the demand of informed consent for enabling the patient to weigh benefits against risks to reach a balanced rational decision. Hence, providing the information about treatment and its benefits separate from risk information, the latter given in form of a list, is often a serious mistake. Another nuisance is unnecessary risk information for repeated interventions (radiological procedures with contrast injection), interdisciplinary overlap (e.g., transfusion risks explicated by surgeon and anesthetist), or repeating the information to the already informed patient (Zech et al., 2015a). Nocebo effects are based on learning, and learning is deepened with repetition (Colloca et al., 2018).

Moreover, it is important to avoid or correct false expectations. The risk of developing myocardial infarction might be of heightened concern in a patient who has recently experienced the death of an acquaintance who died from a heart attack after unsuccessful attempts of resuscitation. This patient should be told that the situation is completely different when occurring in the hospital environment. As soon as any changes are observed on the ECG monitor, ECG and laboratory values are checked, and if the suspicion of an eminent MI is confirmed, immediate treatment is initiated, such as radiological stent implantation, balloon dilatation or lysis, or bypass surgery. Importantly, outcome of myocardial infarction is completely different, when occurring outside versus inside a hospital (Fredriksson et al., 2010).

Further examples are medical lingo, where the understanding of doctors and patients may differ significantly. “Shot!” when taking an X-ray picture. “Reduce the dead space!” is the request for a young assistant to remove part of the breathing tube when the patient has started to breath by himself again using a term from physiology. To a patient the word “dead” in this expression has a disturbing meaning “Label this with “Emergency. Life threat.”” may shock a patient with a minor injury not knowing that the doctor just wants to speed up the processing of the blood sample in the lab.

## Negative Suggestions

There is a tendency in the literature recently to classify all negative aspects of communication as nocebo effects. For a better understanding of the impact of such doctor-patient communication it is helpful to add and distinguish additional models for further explanation. They are not in competition but complementary. Beside expectation and conditioning dependent on learning, behavioral research for instance suggests innate reactions to stimuli.

### The Trance/Suggestion Model

“The importance to recognize that patients before surgery behave as though hypnotized,” an article in 1962 by gynecologist and hypnotherapist David Cheek draws focus to the “different” reactions of patients to everyday situations (Cheek, 1962). Signs of a natural trance state can be recognized in patients during medical emergencies, i.e., situations that elicit stress and fear, where humans (as well as higher animals) enter a different state of consciousness. Among other reactions this state is characterized by heightened attention and an increased suggestibility (Hansen et al., 2010). Clearly, this model appreciates the important observation that patients often behave “differently” when confronted with a medical situation. They become highly susceptible to verbal and nonverbal input and tend to project any information they can grasp onto themselves. Much can be learned from hypnotherapy about this altered state of consciousness and about the nature and use of suggestions (Barber, 1978). In contrast to placebo/nocebo research, the role of the (altered) state of the patient is emphasized in the trance/suggestion model. The effectiveness of a suggestion and the mental and physical reactions that it elicits are much stronger in the trance state than in everyday situations. Mentioning “lemon” leads to some increase in salivation; in hypnosis the suggestion of the image of a lemon can induce tremendous saliva production and secretion. The (trance-) state-bound effect of a suggestion is much more pronounced than the trait-bound effect according to suggestibility scales. Especially in clinical situations the scores of Harvard Group Scale of Hypnotic Susceptibility (HGSHS) or Stanford Scale of Hypnotic Susceptibility (SSHS) lose the impact they typically cause in experimental or psychotherapeutic settings (Montgomery et al., 2011). Given a sufficiently severe emergency situation with stress and pain, every patient enters this natural trance state as an innate protective reaction, and any suggestion is likely to exert a strong mental and physical effect. This opens the door to many adverse effects, but also presents an opportunity to modulate involuntary body functions (Cheek, 1962; Barber, 1978; Jacobs, 1991; Hansen et al., 2010).

The other characteristic of the trance state that is of tremendous clinical importance is focused attention. After extubation an anesthesiologist turned from the patient to the nurse telling her “Don’t throw this tube away! Give it to sterilization.” The young female patient opened her eyes and shouted: “No sterilization! No sterilization!” (Hansen and Bejenke, 2010). In normal life or in a class of students no such reaction is seen. But in this medical situation and natural trance the patient is highly attentive with a high propensity to refer anything to himself. That makes unmindful conversations, or even discussions at the patient’s bed about the problems of other patients, so dangerous. Like dry sponge patients, also with eyes closed and presumably sleeping or in coma, may grasp any information as relevant for themselves. With luck we may become aware of such misunderstandings and can dispel them. These examples provide insight into the altered attention and perception by patients in medical situations and draw our focus to the need to adapt our communication accordingly (Hansen et al., 2010).

In addition, the negative effects of sentences like “Don’t worry” or “You don’t have to be afraid” are better explained by elicited strong images that cannot be not erased by negation than by expectation. Actually, positive expectations and placebo instead of nocebo effects were to be expected from these phrases. Different principles are active in placebo/nocebo and hypnosis/trance. The nature of analgesia produced by hypnosis and placebo suggestions is inherently different (McGlashen et al., 1984; Price and Barrell, 2000; Kupers et al., 2005). A structured literature search in PubMed with the terms “hypnosis AND placebo” (discarding especially papers on pharmacological “hypnosis” and “placebo-controlled” hypnosis studies) over the last 20 years resulted in 18 suitable articles (Page et al., 2001; Ploghaus et al., 2003; Lankton, 2013; Frischholz, 2015). For instance, the analgesic effect of a placebo can be blocked by naloxone, an opiate antagonist, while that of a hypnotic suggestion cannot (Amanzio et al., 2001). Brain imaging shows that hypnosis and placebo/nocebo effects have different bases and representations in the brain (Parris, 2016). Hypnotic suggestions have shown beneficial effects, both when given preoperatively (Defechereux et al., 2000; Häuser et al., 2016) or during general anesthesia (Rosendahl et al., 2016). Hence it is reasonable to assume that by the same mechanism, inadvertently, negative effects can also be induced. In for ethical reasons unrepeatable experiments Levinson demonstrated recall and reactions of negative suggestions given during surgery under general anesthesa (simulation of a ventilation incident) in 8 out of 10 patients (Levinson, 1965). In contrast, placebo and nocebo effects and their mechanisms in unconscious patients are not known.

### Examples of Negative Suggestions

**FIGURE 3:**
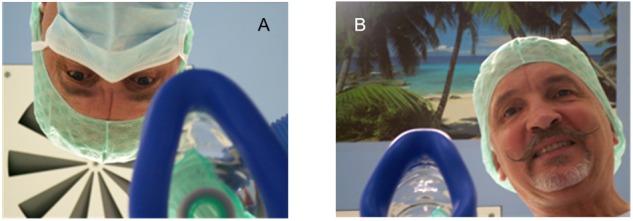
Patient’s view of anesthesia induction. **(A)** Overhead with mask and with air conditioning at the ceiling. **(B)** Face-to-face with poster at the ceiling.

Some suggestions evoke expectations and placebo/nocebo effects, while others have direct effects by addressing inner images. Examples are nonverbal suggestions like the sequence of lamps and air-conditioners a patient sees when transported in the hospital in strict supine position. An upright position avoids this weakening effect (Zech et al., 2019). Similarly, the standard over-head position of the doctor during induction of anesthesia (Figure 3A) is frightening with somatic reactions (Hansen et al., 2010). Induction while situated face-to-face can eliminate this weakening effect, which is also possible during pre-oxygenation with a mask. The poster at the ceiling in Figure 3B is a suggestion for dissociation to a comfortable “safe place,” since any place is better than a dental chair or an operation room.

In the recovery room a patient may lie stock-still in his bed after the instruction “You can call us again when you feel sick or like vomiting” which he understood literally –not unlikely for the trance state – as not being allowed to ask for anything until he feels sick. The words “We’ll put you to sleep” of the anesthetist might remind a patient when his dog was euthanized. “I will be back to see you tomorrow, if you are still here” can sound quite disturbing, when not reflected that he will be moved to another ward. An unrelated thoughtless conversation about a football team that probably “will not make it” can lead to the fearful question of a patient: “Doctor, do you think I will make it?”

### Further Negative Effects Beside Nocebo

Negative effects in patients can be also induced by misunderstandings, uncertainties, lies, enforcement of passivity and unmatched levels of communication that hardly can be explained by expectation and nocebo (Hansen and Bejenke, 2010; Hansen, 2011; Häuser et al., 2012; Zech et al., 2014). Lies like “This will not hurt” or “This won’t take long!” have negative effects by disrupting the therapeutic relationship, although positive expectations and thus placebo effects should be expected. Misunderstandings should be avoided. “Let me cut this!” is frightening when not followed by the explanation “- the suture.” A doctor that wants to explain to the team his late arrival due to a traffic jam shouting “A disaster!” really can upset the patient under treatment. How many patients know that “We tested for tumor, the result was negative” are good news? Announcing local anesthesia with the words “I’ll give you an injection so you won’t feel anything” is misleading information since transmission of pain and sensation involve different types of nerve fibers and are differentially blocked by local anesthetics. “Do you already notice anything?,” “Let us try this” or “Try to take the pills regularly” projects the disturbing suggestion of uncertainty and the impression that the therapist doesn’t know what is going to happen, or does not believe that his patient will take the pills. Unmatched levels of perception or solution strategies are the basis of the following negative examples. A nurse to a patient crying after a diagnosed cancer: “Just pull yourself together!”. The response of a doctor to a patient’s complaint “I have this awful pain in the chest.”: “Don’t worry, the lab result is ok.” As important and positive as this message is, he forgot that he is focused on a clinical picture, while the patient has a sickness as an existential experience. He must recognize the fear that lies behind that sentence, the unspoken question “Am I suffering a myocardial infarction? Am I going to die?,” and address it.

“Try to calm down,” “Just relax!,” “Pick a nice dream!” are insufficient instructions, and are perceived negatively by demonstrating to the patient his inability to comply with the task. Instead, the desired tasks can be achieved by positive suggestions. “Just breathe normally” (Schenk, 2008) sets a wrong focus. “Just stay away from saturated fats” is an oversimplification that reduces weight control to fat uptake and ignores the impact of sugar or sports.

One of the worst negative suggestions in clinical practice is a request for passivity often required from a patient. “Be patient! All you have to do is keep still. Let us just do our job. You can put your mind at rest, we have done this a thousand times.” The difference when a patient is actively participating in the therapy is evident and very impressive in the case of awake craniotomies without sedation (Hansen et al., 2010; Seemann et al., 2015a; Zech et al., 2018). Here, the awake and guided patient contributes to his own brain surgery (deep brain stimulation or tumor resection in the vicinity of eloquent or motoric areas) by dissociation to, and creation of, a “safe place” and “reframing” of noises (e.g., drilling) and other disturbing sensations, thus avoiding pharmacologic sedation and allowing for unimpaired intraoperative neurological testing. Strengthened by appreciation and the experience of self-sufficiency they go into subsequent chemo- and radiation therapy.

### Using Positive Suggestions

The suggestion of an image of flowing water can promote peristaltic. Conjuring the image of holding the injured hand into fresh fallen snow or ice-cold water stops the bleeding because of vasoconstriction. This can hardly be explained by expectation or as a placebo effect, since generally the patient does not know of this connection or the fact that body functions are structured along inner images. Often positive communication is confused with withholding negative information, lies, or whitewash. Rather, it should consist in pointing out the positive aspects that exist. When informing a patient about the possibility of postoperative pain and the request to inform the nurse about it the distressing word “pain” can be exchanged by the word “pressure,” and a positive suggestion about “healing” can be communicated by the statement: “When you feel a pressure under the bandage then tell us, because it shows that the healing has already begun.” The latter is not a lie but the truth. Actually, what the patient feels is the action of leukocytes and their cytokines causing vasodilatation, edema and pain. Those are the symptoms of the healing process, a fact that should be communicated to the patient. Thus, the distressing word “pain” can be exchanged by more neutral suggestions, or can be set into a positive context, like healing, or protection from too early postoperative mobilization, a process described as “reframing.” The meanings of “pain” range from protection as its natural function to suffering. Sometimes it is helpful to point out to the patient his “interpretive sovereignty,” thus giving back control.

In an interesting study Choi et al. have compared the use of a pain scale and a comfort scale in women after cesarean section (Chooi et al., 2013). The remarkable result was that although the percentage of patients with pain was similar, later on the pain score was significantly lower for both rest and in movement, and patients had less demand for analgesics, when the comfort scale was applied. In addition, these patients described their experience as less bothersome or less unpleasant, and viewed their surgery as “wound healing” rather than as “tissue damage.” It can be expected that the latter has a long-lasting impact on their memory, feelings and attitude toward cesarean section and even their child. Therefore, the wide use of pain scores in pain medicine and oncology should be reconsidered. Often the argument is that you have to ask about “pain” to get the necessary information. Actually, after a question “Are you comfortable. Can we do anything for your?” a patient suffering from pain will declare his pain. For the negative effect it makes quite a difference whether the stressful word “pain” comes from the outside via medical authority, or well integrated from the patient himself.

Similarly, “bleeding” is a negative signal to the patient, although it also has the aspect of cleaning the wound and recruiting coagulation factors and platelets in order for a scab to develop and healing to take place. Such explanations to the patient can reframe the negative suggestions elicited by the words “pain” and “blood” away from the nowadays common ideas of patients that something must have gone wrong or a failure must have occurred and somebody has to be sued (Hansen and Bejenke, 2010).

## Therapeutic Relationship

A further lesson can be learned from hypnotherapy: The potency of suggestions is dependent on the context, namely the sum of the individual history and predispositions of the patient and the context of the relationship (Hansen, 2010; Hansen and Bejenke, 2010; Arnon, 2016). When I am strolling along and get hit on my back, I turn around and it’s a colleague I haven’t seen for years, I am happy and feel no pain. When I turn around and instead see a bold skinhead, it hurts like hell and I run away. Completely different reactions to the same event. Therefore, the quality of the relationship between doctor and patient is the most decisive determinant between the injurious impact of negative suggestions and the beneficial impact of positive ones. This corresponds to the finding that the therapeutic relationship is the central effect factor in psychotherapy (Grawe, 2000; Wampold, 2015), and the experience with hypnosis in medicine (Jozsa, 2011; Arnon, 2016; Ebell, 2017). Accordingly, the best protection of patients against harm from risk information is a trusting relationship, and by the way also against being sued. Judges confirm that to suffer harm usually is not a sufficient condition for a patient to file a suit. Only when harm is accompanied by a communicative failure, i.e., the lack of a positive relationship or of contact and communication after the fault occurred, may patients go to court (Seemann et al., 2015b).

Any understanding of the practice of medicine, where the doctor neutrally explores and observes the symptoms of a patient, objectively provides a diagnosis, and after informed consent starts a therapy with purely inherent effects and side effects to finally watch the outcome as an uninvolved external observer should be abandoned. The way he asks about symptoms will have an impact, and the way he communicates a diagnosis will shape the course of the disease for years. The choice of words and the clinical setting when providing information on therapeutic benefits and expected side effects will impact the patient’s emotional and physical response. Through his communication the therapist directly affects the patient, his symptoms, disease progression and ultimately its outcome. A concept for a positive, “therapeutic” communication is given in Table 2. Examples of clinical applications are listed in Table 3.

**Table 2:** A concept for therapeutic communication.

(1) Knowledge, recognition and avoidance or neutralization of nocebo effects and negative suggestions
(2) Positive communication: positive expressions instead of negations no lies, whitewash, or non-disclosure to address existing positive aspects to address the psychological basic needs (basic communication) anouncement and addition of meaning to any intervention suggestion of positive expectations positive suggestions and interventions (e.g., dissociation, reframing)
(3) Utilization of the focused attention and suggestibility of the stress-induced natural trance state
(4) Development of a trusting, encouraging therapeutic relationship

**Table 3:** Clinical applications of therapeutic communication.

Emergency medicine	Jacobs, 1991; Hansen et al., 2010
Treatment of children	Zech et al., 2015b
Risk information for informed consent	Seemann et al., 2015b; Zech et al., 2015a; Colloca, 2017
Preparation for surgery	Hansen, 2010; Hansen and Bejenke, 2010; Häuser et al., 2016; Kendrick et al., 2016
Care during local or regional anesthesia	Defechereux et al., 2000; Hansen et al., 2013; Seemann et al., 2015a; Zech et al., 2018
During general anesthesia	Rosendahl et al., 2016
Pain therapy, Psychooncology	Jensen and Patterson, 2014; Kendrick et al., 2016; Facco et al., 2018

## Author Contributions

EH conceived and wrote the manuscript. NZ participated in literature search and discussion. Both authors revised the manuscript and approved the final manuscript for submission.

## Conflict of Interest Statement

The authors declare that the research was conducted in the absence of any commercial or financial relationships that could be construed as a potential conflict of interest.
